# Polarisation reconfigurable anisotropic dielectric resonator antenna

**DOI:** 10.1038/s41598-025-94491-3

**Published:** 2025-04-01

**Authors:** Shadi Danesh, Mohammad Abedian, Mohsen Khalily, Pei Xiao, Rahim Tafazolli, Ahmed A. Kishk

**Affiliations:** 1 5G & 6G Innovation Centres (5GIC & 6GIC), Institute for Communication Systems (ICS), University of Surrey, Guildford, GU2 7XH UK; 2 HID Global, GU10 5EH Farnham, UK; 3 Department of Electrical and Computer Engineering, Concordia University, Montreal, Canada

**Keywords:** Engineering, Electrical and electronic engineering

## Abstract

A novel polarization reconfigurable anisotropic dielectric resonator antenna (ADRA) is presented utilizing a new modulation scheme to exploit the degree of freedom in the polarization domain. The ADRA comprises periodic assembly dielectric resonators with two different dielectrics constant, equal in size, a vertically positioned metal strip, one varactor diode, and six PIN diode switches. The modulation scheme utilizes the tilt angle and axial ratio (AR) of a wireless signal to convey additional information, enabling the realization of different working modes ranging from circular polarization (CP) to nearly linear polarization (LP). The proposed modulation scheme yields significantly better bit error rate (BER) performance and higher spectral efficiency in bits/s/Hz/antenna. Additionally, the paper presents an antenna design capable of generating an arbitrary polarization state, highlighting the system benefits of polarization modulation. Post-fabrication, the proposed approach is validated by comparing simulated and measured results. The proposed antenna provides a total efficiency higher than 93% in the desired frequency bands and consistent gain at approximately 7.64 dBi and 7.09 dBi at 3.8 GHz, with the impedance matching bandwidth ranging from 3.53 to 3.90 GHz and 3.56 to 3.91 GHz fully overlapping across all polarization states for the simulated and measured results, respectively. Experimental results affirm the robust performance of the proposed ADRA.

## Introduction

The swift advancement of wireless communication systems, characterized by multifunctional capabilities, necessitates modern devices with enhanced communication performance to meet the requirements of the new multimedia applications. The escalating number of mobile users and the surge in mobile traffic underscore the need for substantial improvements. Projections indicate a 1000-fold increase in global mobile data traffic over the next decade^1–3^. Spectrum scarcity has emerged as a critical challenge in the microwave frequency bands, impeding the seamless deployment of new wireless services.

Antennas featuring reconfigurable polarization, radiation, and frequency capabilities, with minimal clutter and complexity, provide a robust solution for enhancing the performance of network systems across various objectives. These antennas are adept at mitigating channel interference, showcasing low power consumption, and minimizing system complexity. Their adaptive characteristics contribute to intelligent systems’ development, offering a versatile and efficient solution for diverse applications. In addressing the traffic demands within the desired electromagnetic spectrum and enhancing channel capacity, reconfigurable polarization antennas are pivotal. These antennas can alter a signal’s relative polarization, facilitating effective communication in ultra-dense network scenarios^4^.

On the other hand, significant research efforts have been dedicated to studying dielectric resonator antennas (DRAs). These antennas are an excellent choice for reconfigurable antenna design owing to several inherent features, such as low profile, no surface wave, wideband, various excitation mechanisms, low loss, and geometrical flexibility^5,6^. Compared to microstrip antennas, which suffer from limitations like narrow operating bandwidth and low radiation efficiency, DRAs present enhanced flexibility attributed to their 3D structure, low Q-factor, and absence of metallic loss. Among the three primary DRA shapes, the rectangular DRA stands out as the most common, offering advantages such as providing two different aspect ratios (width/length and height/length) at a desired resonant frequency compared to spherical and cylindrical counterparts^7^. Simultaneously, the anisotropic rectangular dielectric resonator antenna (ARDRA), featuring a rectangular dielectric resonator (DR) prism made of a uniaxial anisotropic dielectric medium, demonstrates notable advancements in antenna radiation performance^8,9^. In other words, unlike the conventional DRA that primarily radiates from the top wall, implementing an ADRA enhances radiation from the sidewalls. This enhancement is achieved by intensifying the magnetic current densities on the sidewalls, resulting in an improved directivity of the ADRA at the fundamental radiating mode^10^. The work in^9^ and^10^ emphasized the critical role of anisotropic materials in controlling radiation and enhancing antenna performance. In^9^, uniaxial anisotropic materials improved boresight directivity by increasing radiation from the sidewalls, leveraging boundary condition effects and magnetic current densities. In^10^, the anisotropic dielectric layers were utilized to achieve precise control over circular polarization and radiation patterns, enabling high transmit/receive isolation (>50 dB) while maintaining consistent radiation efficiency. The proposed design builds on these concepts using anisotropic materials to achieve superior radiation pattern control, polarization versatility and enhanced operational performance.

**Fig. 1:**
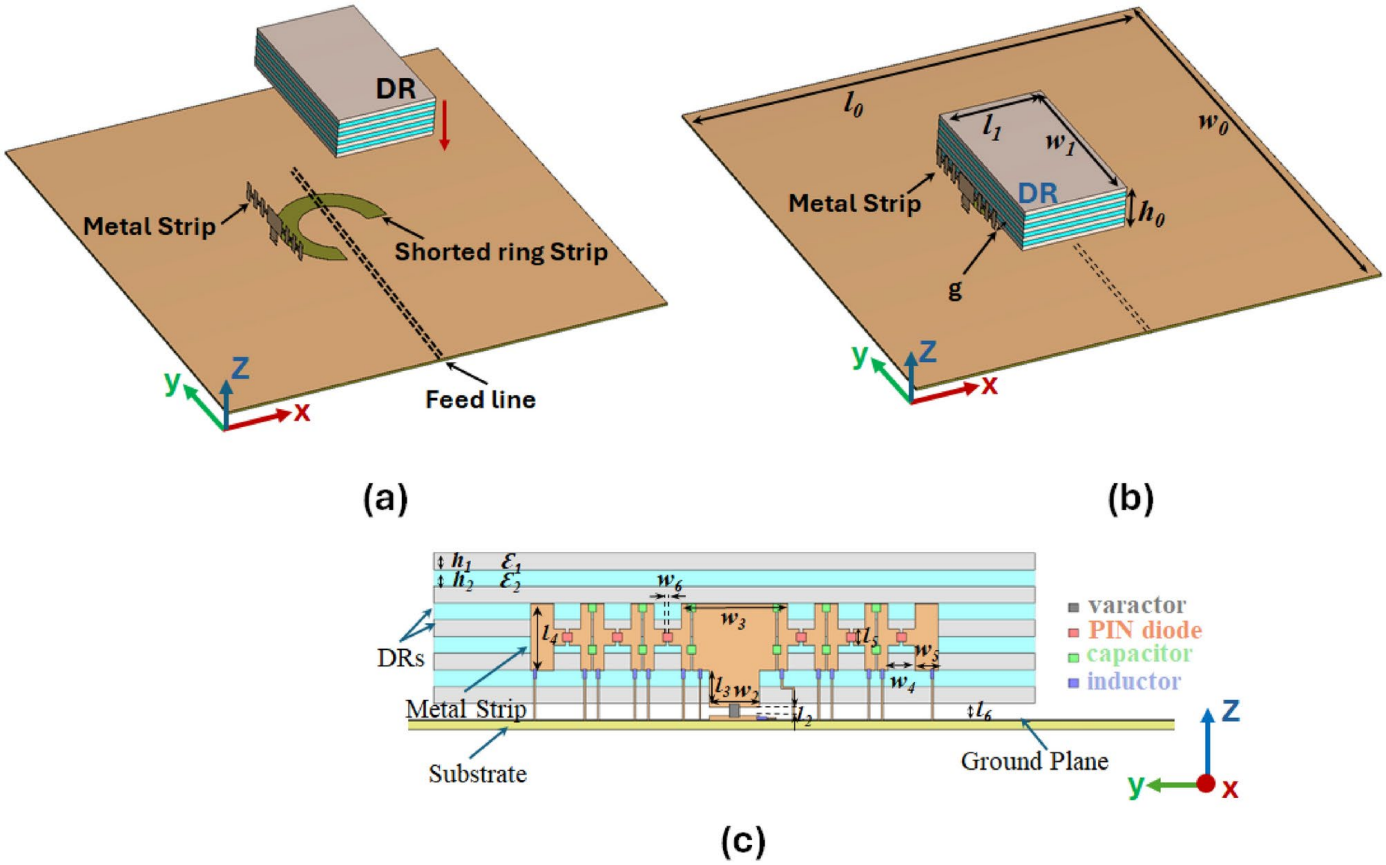
The proposed ARDRA: (**a**) a microstrip shorted ring slot-coupled feeding mechanism, (**b**) The proposed configuration, (**c**) side view (explanation of metal strip adjacent to the DR with a distance g).

Circularly polarized antennas in real-world scenarios can attain an axial ratio (AR) of less than 3 dB^11,12^. Yet, they can never achieve a perfect circular polarization with an axial ratio of 0 dB. Imperfections inherent in practical antennas prevent them from achieving polarization purity for perfect linear or circular polarization. Consequently, elliptical polarization is radiated instead. Any circular polarization becomes elliptical when the magnitudes of the vertical and horizontal polarization signals are unequal and/or there is no longer a 90-degree phase shift between them. Elliptical polarization introduces two more degrees of freedom in the polarization, the tilt angle and AR, which can be utilized to carry additional information. A significant challenge in this domain is achieving dynamic reconfiguration of the polarization state. Presently, existing polarisation reconfigurable antennas can only switch between orthogonal polarization states (e.g., LHCP and RHCP) or transition between linear and RHCP/LHCP and vice versa. Several designs of polarization reconfigurable antennas have been introduced, employing three effective techniques to generate switchable polarization^13–23^. These methods can be realized by utilizing polarizers to alter the polarization^13–15^, introducing a reconfigurable resonator integrated with RF switches^16–19^, and employing a reconfigurable feeding network^20–24^. Polarizers were mainly employed for polarization reconfigurability^13–15^. The design in^13^ integrated a cross-shaped slot and parasitic traces with PIN diodes to switch between LHCP, RHCP, and LP states, primarily targeting waveguide applications. In^14^, a planar antenna with a 4 $\times$ 4 polarizer array of PIN diodes was utilized to toggle between LHCP and RHCP, emphasizing low-profile design and high efficiency for planar systems. An active metasurface of elliptic split rings with PIN diodes is applied to convert LP to CP as a superstrate with horn antennas^15^. In contrast, our approach eliminates the reliance on discrete polarizer arrays or external converters, achieving continuous axial ratio tuning and elliptical polarization through intrinsic reconfigurability of the antenna structure. Such flexibility enables a broader range of polarization states, tilt angle modulation, and advanced communication capabilities, surpassing these designs’ static or discrete-state limitations. Concurrently, numerous dielectric resonator antennas (DRAs) have been explored to advance polarization diversity and reconfigurability^25–31^. Compared to our design, the work in^25–31^ exhibited several limitations. For instance,^25^ was restricted to discrete polarization states without smooth transitions or advanced modulation schemes, while^26^ relied on slow mechanical reconfiguration and lacked support for intermediate polarization states. Similarly,^27–31^ were constrained to specific polarization modes or states (linear or circular), failed to achieve continuous axial ratio tuning or intermediate elliptical polarizations, and did not introduce innovative modulation schemes leveraging polarization characteristics for enhanced communication capabilities, which is uniquely provided with our design.

In^27^, the authors present a dielectric resonator antenna with reconfigurable linear (vertical and $\pm 45^\circ$), circular polarizations (LHCP, RHCP), and broadside and conical beam switching. Still, it lacks intermediate polarization states and modulation capabilities. Similarly,^29^ introduces a liquid-metal-based DRA for discrete polarization states ($-45^\circ , +45^\circ$, and y-axis), but its functionality is limited to fixed states. In contrast, our design enables continuous axial ratio tuning from CP to LP, allowing full elliptical polarization control and seamless transitions using varactor diodes. Additionally, our novel modulation scheme utilizes tilt angle and axial ratio for transmitting supplementary information, making it a highly adaptable and advanced solution for wireless communication.

To the best of our knowledge, there is currently no existing literature on antennas capable of reconfiguring to achieve an arbitrary polarization state through manipulating tilt angle and axial ratio.

In this work, we delve into an additional dimension of the polarization domain where the polarization of a radio wave is harnessed to convey information-bearing signals. By discerning the rotation direction of an elliptically (or circularly) polarized electric field, along with considering the amplitude ratio and phase differences of the horizontal and vertical components, distinctions in the polarization status of radio waves can be achieved. Elliptical polarization enhances the modulation order, given that the tilt angle and axial ratio (AR) carry additional information. This paper tackles the spectrum/capacity limitations challenge by unlocking degrees of freedom in the polarization domain through a groundbreaking cross-disciplinary approach, resulting in a novel modulation scheme. In particular, we introduce Elliptical Polarization Modulation (EPM) as a novel approach to enhance the spectral efficiency of systems. Through the incorporation of a varactor diode and six p-i-n diodes positioned on a vertical parasitic element, a promising capability for continuous tuning of the axial ratio (AR) and tilt angle is achieved. By simultaneously exciting two orthogonal resonant modes, $TE^{111}_x$ and $TE_y^{111}$, via a circular coupling slot, Circularly Polarized (CP) waves are generated. The polarization reconfigurability is implemented by selectively activating and deactivating the p-i-n diodes, allowing for the tuning of varactor capacitance across its entire range. This principle facilitates the realization of various polarizations in terms of AR and tilt angle throughout the entire frequency tuning range. The proposed ADRA design is particularly well-suited for dense, high-capacity environments, such as urban 5G/6G networks, where spectrum efficiency is paramount. The reconfigurable polarization feature enables adaptive signal transmission, dynamically adjusting to changing channel conditions, and ensuring seamless integration into systems that demand high data rates and low latency.

## Configuration and operation of polarization reconfigurable ARDRA

Elliptical polarization modulation can introduce potential degrees of freedom. Thus, the objective is to explore the potential of conveying extra information bits within a given modulation scheme through the polarization state of the wave. Typically, the polarization of a propagating wave is characterized by two components: the axial ratio (AR) and the tilt angle. Existing reconfigurable polarization antennas in the literature primarily focus on switching between orthogonal polarizations or transitioning between linear and CP^13–31^. However, this paper proposes an approach to continuously reconfigure the polarization components simultaneously. To achieve this objective, a calculation template is introduced and developed, enabling the computation of the AR and tilt angle of the radiated wave for a given antenna structure. In developing such an antenna, paramount considerations include an easy fabrication process and a cost-effective design. An antenna is developed, designed, and fabricated according to these essential criteria. The ensuing results are comprehensively analyzed to assess the performance and efficacy of the proposed design.

### Template post-processing development

To achieve the discussed design goals, developing a code capable of accurately evaluating the antenna’s tilt angle and axial ratio (AR) of an antenna is imperative. The code should facilitate a comprehensive analysis of the design and post-processing, be easy to optimize, and ensure swift execution with an adequate number of sampling points. The template developed adheres to these features, as elucidated in the following discussion. In assessing the polarization states of a propagating wave, three types-linear, circular, and elliptical-exist for harmonic fields based on the shape of the wave’s trace. The representation and evaluation of any polarization state can be achieved if the two orthogonal fields, linearly polarized ($E_x$, $E_y$), of that propagating wave are known. Generally, these orthogonal fields ($E_x$, $E_y$) are complex numbers representing amplitude and phase. The magnitudes of the orthogonal fields may differ, and they may be out of phase by an angle $\delta _L$. Utilizing these notations, the polarization states of the wave can be effectively evaluated.

- If $\delta _L$ = 0 or n$\pi$, then the field is linearly polarized.
- If $\delta _L$ = $\dfrac{\pi }{2}$ and $|E_x|$ =$|E_y|$, then the field is circularly polarized.
- In the most general case, the polarisation is elliptical.

**Fig. 2:**
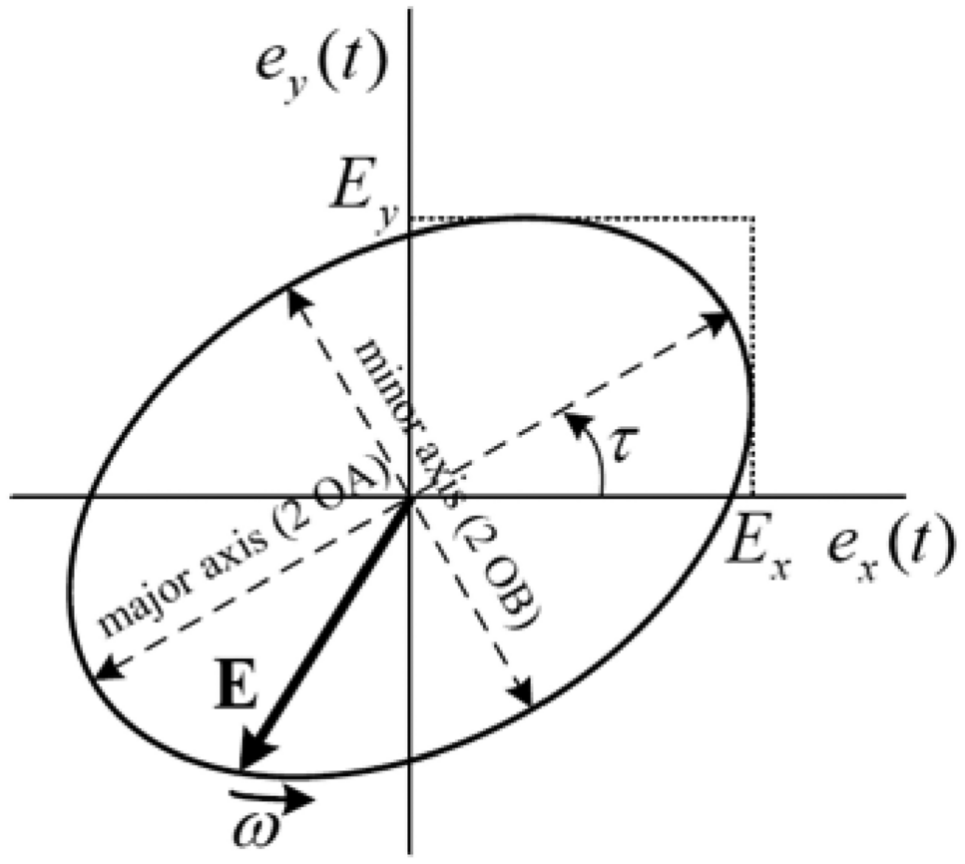
The basics of the polarization ellipse.

In this paper, the equations for the most general case, namely elliptical polarization, are discussed and utilized to calculate tilt angle and axial ratio (AR). These equations are the foundation for developing the template^32^. The AR and the tilt angle of an ellipse can be derived by knowing two orthogonal components on the ellipse ($E_y$, $E_x$ in Fig. 2). Once these two components are known as the major and minor axis of the ellipse can be derived from equations (1) and (2) as follows:

1 $$\begin{aligned} OA= & \sqrt{\frac{1}{2} \left[ E_x^2 + E_y^2 + \sqrt{E_x^4 + E_y^4 + 2 E_x^2E_y^2 \cos (2\delta _L)} \right] } \end{aligned}$$

2 $$\begin{aligned} OB= & \sqrt{\frac{1}{2} \left[ E_x^2 + E_y^2 - \sqrt{E_x^4 + E_y^4 + 2 E_x^2E_y^2 \cos (2\delta _L)} \right] } \end{aligned}$$

Knowing the major and minor axis of an ellipse, the AR and tilt angle $(\tau )$ can be calculated from equations (3) and (4) as follows:

3 $$\begin{aligned} (\tau )= & \frac{1}{2} \arctan \left[ \frac{2 E_x E_y}{E_x^2 - E_y^2} \cos (2\delta _L) \right] \pm \frac{\pi }{2} \end{aligned}$$

4 $$\begin{aligned} AR= & \dfrac{major axis}{minor axis} = \dfrac{OA}{OB} \end{aligned}$$

The challenge lies in integrating these equations into a full-wave simulation for thorough design analysis and optimization to achieve the desired polarization state. CST Microwave Studio was used for this purpose. The template-based post-processing feature in CST enables the implementation of any equation. Two orthogonal field probes were defined in the simulation at the boresight in the far-field of the antenna. The phase and amplitude of the orthogonal fields can be monitored, and once $\delta _L$, $|E_x|$ and $|E_y|$ are determined from the field probes, codes can be developed accordingly. Consequently, for any antenna design, the AR and tilt angle can be calculated, and the impact of design parameters can be systematically monitored. Furthermore, the design can be optimized for the desired AR and tilt angle.

### Methodology

To exert control over the polarization, AR, and tilt angle of the propagating wave emitted by an antenna, it is imperative to manipulate the components of the electric field in the plane of the antenna (i.e., ($E_x, E_y$)). The proposed approach employs a varactor diode alongside six P-I-N diodes mounted on a vertical parasitic metal element. To validate this approach, an Anisotropic Rectangular Dielectric Resonator Antenna (ARDRA) parallel to the vertical element is designed on a grounded Rogers 4003 substrate with a thickness of 0.508 mm. To mitigate the presence of significant ripples in the far-field patterns, a ground plane larger than one wavelength in dimensions is selected. The dimensions of the proposed antenna are depicted in Table 1. Additionally, a pair of orthogonal field probes ($E_x, E_y$) is developed to assess the impact of the vertical parasitic element on the antenna’s performance. A uniaxial ADRA is achieved by stacking dielectric sheets with two different dielectric constants when the sheets’ thickness is sufficiently small (less than one-tenth of the wavelength)^33^. In this study, each adjacent sheet possesses a different dielectric constant but equal thickness, as illustrated in Fig.1(c). In other words, the uniaxial anisotropic structure is introduced by sandwiching the DR with lower permittivity between the one with a higher dielectric constant. This arrangement forms a permittivity tensor with parameters $\epsilon _x = \epsilon _y > \epsilon _z$, resulting in an equivalent homogenized permittivity tensor, as described in^9^.

5 $$\begin{aligned} \epsilon _x = \epsilon _y = \dfrac{\epsilon _1 \epsilon _2 (d_1+ d_2)}{\epsilon _1 d_1 + \epsilon _2 d_2} , \epsilon _z = \dfrac{\epsilon _1 d_1 + \epsilon _2 d_2}{d_1 + d_2} \end{aligned}$$

**Fig. 3:**
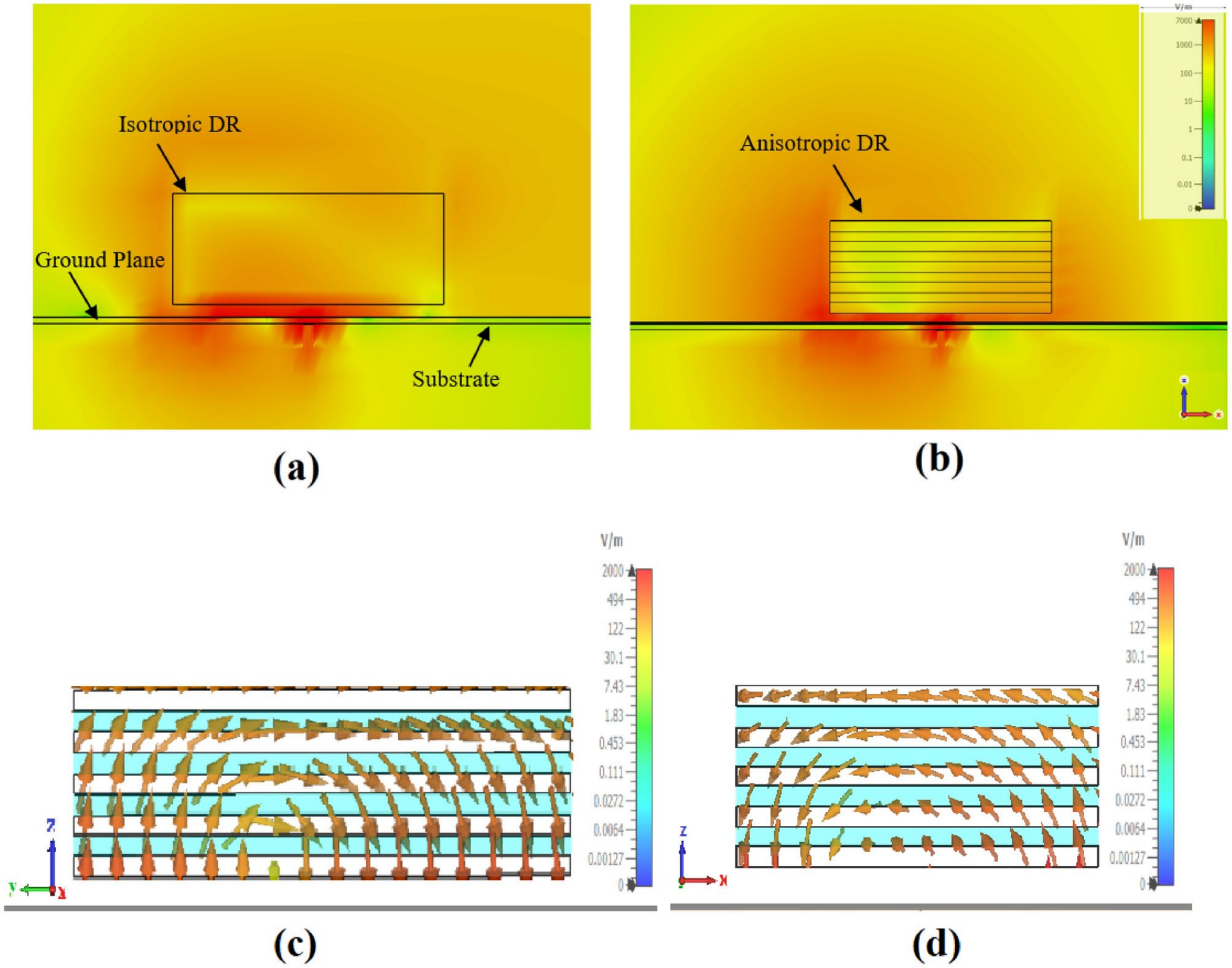
Simulated radiation intensity and excitation modes of polarization reconfigurable DR. (**a**) radiation intensity of Isotropic DRA (IDRA) (Y-direction), (**b**) radiation intensity of Anisotropic DRA (ADRA) x(Y-direction), (**c**) $TE_x^{111}$ mode, (**d**) $TE_y^{111}$ mode.

It is noted that the maximum anisotropic effect is achieved when ${d_1}$ = ${d_2}$^32^. Furthermore, a notable improvement in electromagnetic leakage from the sidewalls of the ADRA, compared to the top wall, can be achieved by reducing the value of $\epsilon _z$. For instance, reducing the ratio $\dfrac{\epsilon _z}{\epsilon _x}$ from 1 (indicating an isotropic medium) to 0.2 (indicating an anisotropic structure) has resulted in a significant decrease in the radiation intensity from the top wall compared to the sidewalls^9,10^. Based on the DWM method elaborated in^9^, the proposed ARDRA was modeled and designed to operate at 3.8 GHz. Each sheet is constructed using ECCOSTOCK^®^ HiK dielectric material, characterized by a loss tangent ($\tan \delta$) of 0.002 and an equal thickness of 1 mm. Notably, two relative permittivities, $\varepsilon _r = 15$ (for layers 1, 3, 5, 7, 9) and 2 (for layers 2, 4, 6, 8), are employed. Consequently, the effective permittivity tensor, calculated using Equation (5), is determined as $\varepsilon _x = \varepsilon _y = 8.5$, $\varepsilon _z = 3.53$. Figure 3a,b indicates altering the radiation intensity from the top wall to the sidewalls.

**Table 1:** The proposed ARDRA dimensions.

Parameters	Value (mm)	Parameters	Value (mm)
$w_0$	100.00	$l_0$	100.00
$w_1$	36.00	$l_1$	22.00
$w_2$	3.00	$l_2$	0.50
$w_3$	6.40	$l_3$	2.20
$w_4$	1.60	$l_4$	4.00
$w_5$	1.40	$l_5$	1.00
$w_6$	0.20	$l_6$	1.00
$h_1$	1	*g*	3
$h_2$	1	-	-

Fig. 1 shows the configuration of the proposed antenna. It consists of two types of dielectric resonators, stacking dielectric sheets with two different dielectric constants ($\epsilon _{r1}$ = 2 and $\epsilon _{r2}$ = 15) (9 layers total). These layers are constructed using ECCOSTOCK®  HiK dielectric material, each with a uniform thickness of 1 mm and a low-loss tangent ($\tan \delta$ = 0.002). The structure also incorporates a vertical metal strip positioned 3 mm from the ADRA, on which one varactor diode SC-79 (Model: SMV1232-079LF) and six AlGaAs Beamlead P-I-N diodes (Model: MA4AGBL912) mounted on the metal strip to enable polarization reconfigurability. The dielectric resonator block is mounted on a ground plane, fabricated using a Rogers 4003 substrate. A microstrip line of width 0.9 mm and a length of 67.5 mm on the bottom side of the dielectric substrate couples to the shorted ring slot, which in turn excites the resonant modes of the ADRA, ensuring efficient energy transfer and proper excitation of the desired polarization states. To enhance the impedance bandwidth, an air gap, $l_6$ = 1.00 mm, is introduced beneath the dielectric resonator block. This air gap reduces the effective permittivity and supports improved radiation characteristics. The varactor diode adjusts the voltage, thereby varying its capacitance to control the phase and amplitude of the orthogonal electromagnetic fields within the ADRA. This variation allows the antenna to achieve polarization reconfigurability across various states.

**Fig. 4:**
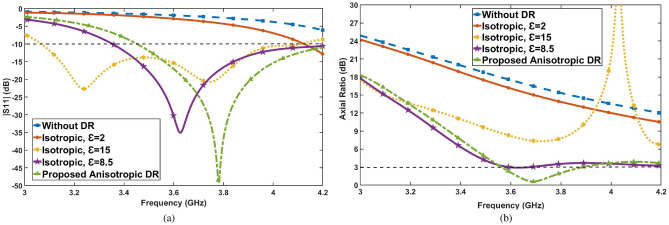
Simulated (**a**) |*S*11| and (**b**) AR of the Isotropic DRA (IDRA) and Anisotropic DRA (ADRA).

To achieve the CP antenna, the shorted microstrip line coupling to the ring slot feeding mechanism (refer to Fig. 1a) is introduced to excite the ARDRA orthogonal modes of $TE_x^{111}$ and $TE_y^{111}$, as illustrated in Fig. 3c and d, respectively. The proposed configuration has been shown in Fig. 1b. To shed some light on the proposed antenna design considerations, a comparison is made between an isotropic DRA with dielectric constants of $\epsilon _r$ = 2, 15, 8.5 and the proposed Anisotropic DRA (ADRA) in terms of the impedance and AR bandwidth. As depicted in Fig. 4, while the utilization of anisotropic DR affects both impedance (Fig. 4a) and AR bandwidths (Fig. 4b), it notably impacts the AR bandwidth. Therefore, compared to isotropic DRs, the proposed ADRA offers an increased 3-dB AR bandwidth of approximately 9.3$\%$. Furthermore, the gain improvement has been obtained using ADRA with a peak gain of about 7.64 dBi, compared to the isotropic DRA with a peak gain of around 6.57 dBi. Notably, the transformation of the DRA structure into ADRA leads to changes in the two excited modes inside the DRA and orthogonal degenerate modes of $TE_x^{111}$ and $TE_y^{111}$ are excited within the ADRA. The simulated E-field distributions confirm a 90-degree phase difference between the orthogonal degenerate modes inside the ADRA at the resonant frequency. For linear polarization, only the $TE_x^{111}$ mode is observed to be excited within the ADRA dielectric. By activating all diodes, the orthogonal degenerate modes responsible for circular polarization (CP) are suppressed, allowing the antenna to transition to a nearly linear polarization (LP) state, where the $TE_x^{111}$ mode remains dominant.

It is worth noting that using an anisotropic dielectric resonator (DR) not only improves the circular polarization (CP) axial ratio but also causes a significant shift in radiation by reducing $\epsilon _z$ from 15 (isotropic) to 3.54 (anisotropic). As shown in Fig. 3b, most of the radiation occurs from the sidewalls, as the magnitude of $E_z$ is strengthened compared to $E_y$^9^, and the radiation intensity of Isotropic DRA, shown in Fig. 3a. This sidewall radiation phenomenon allows one to control the phase and amplitude of the orthogonal fields by introducing a reconfigurable structure close to the proposed Anisotropic DRA (ARDA). Therefore, one varactor diode and six P-IN diodes mounted on a vertical strip are employed to achieve a polarization reconfigurable antenna capable of addressing an arbitrary polarization state. In such a way, by controlling the states of the diodes, several different polarization states can be realized. Based on reconfigurable polarization antenna design, an ADRA has been designed and simulated, featuring a nine-layer Dielectric Resonator (DR) block with two distinct dielectric constants, $\epsilon _{r1}$=2 and $\epsilon _{r2}$=15. Additionally, the design incorporates one varactor diode and six P-I-N diodes mounted on a metal strip (located on the left side of the ARDR with a distance of g = 3 mm), as illustrated in Fig. 1c.

Firstly, the varactor diode is set at its maximum value (4.15 pF) to modulate the Axial Ratio (AR)and the corresponding tilt angle. Table 2 presents all 18 possible states, where the AR and tilt angle are simulated at 3.8 GHz. The varactor diode and the six P-I-N diodes serve as switches for altering the types of polarization. The impact on the antenna’s matching, resulting from variations in the physical length of the strip, is detailed in the table. All other parameters were kept constant. Notably, for a given strip size, the desired operating frequency ranging from 3.56 GHz to 3.91 GHz remains constant and is independent of the strip size. The CP waves can be realized when the varactor and all switches are OFF. In this state, the antenna has a wide impedance bandwidth from 3.41 to 4.2 GHz with an AR bandwidth of 330 MHz from 3.54 to 3.87 GHz. In Table 2, in S_1 and S_2 states, the varactor is OFF, and ON in other states. The table shows that the AR and tilt angle can be adjusted by increasing the strip length (when diodes are ON). In other words, the AR can be tuned continuously from 2.17 to 24.7 dB, while the tilt angle exhibits a changing range from 82.76$^{\circ }$ to 114.5$^{\circ }$. This phenomenon arises from altering the electric field components ($E_x$, $E_y$). In fact, as the length of the strip increases, the $E_y$ component decreases due to an augmented effective resonator dimension in the x-direction.

**Table 2:** Simulated Results of the Polarization-Reconfigurable ADRA when Varactor Capacitance Value is 4.56 pF.

Status	0 = OFF, 1 = ON	Tilt Angle	AR (dB)	BW (GHz)
$S_{1}$	000000	95.65	2.17	3.41–4.20
$S_{2}$	111111	106.84	3.67	3.27–3.92
$S_{3}$	000000	86.91	4.76	3.09–3.91
$S_{4}$	100000	84.76	5.85	3.05–3.94
$S_{5}$	000100	87.24	6.37	3.05–3.94
$S_{6}$	100100	85.84	7.35	3.00–4.02
$S_{7}$	110000	82.76	8.19	3.50–4.20
$S_{8}$	000110	91.32	9.24	3.00–4.02
$S_{9}$	110100	85.21	10.07	3.43–4.02
$S_{10}$	100110	91.05	10.38	3.41–4.11
$S_{11}$	000111	102.07	11.85	3.39–3.95
$S_{12}$	100111	102.85	13.48	3.42–3.96
$S_{13}$	110110	92.69	14.17	3.40–4.02
$S_{14}$	111000	89.33	16.32	3.53–4.07
$S_{15}$	110111	105.99	17.20	3.42–3.97
$S_{16}$	111100	94.14	19.98	3.49–4.09
$S_{17}$	111111	114.50	21.31	3.41–3.97
$S_{18}$	111110	103.71	24.70	3.44–3.98

## Results and discussions

**Fig. 5:**
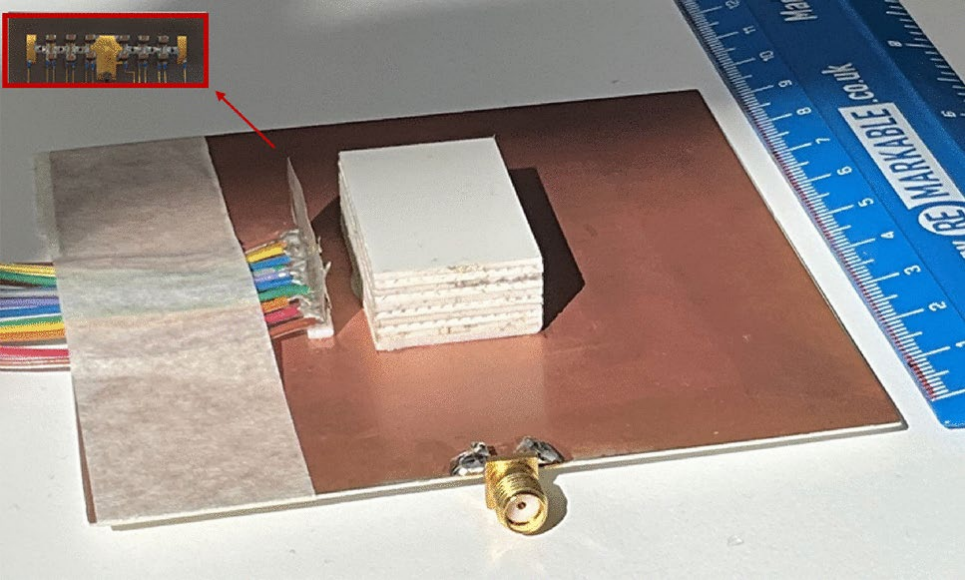
The prototype of the proposed polarization modulation ADRA, parasitic strip is shown in the inset up-left of the figure.

A prototype of the proposed polarization modulation ADRA, as depicted in Fig. 5, is fabricated, assembled, and then measured to verify the proposed antenna performance. The air gap ($l_6$) is realized using a ROHACELL HF foam layer with a dielectric constant close to the free space ($\epsilon _{r}$ $\simeq$ 1.04), which also acts as a mechanical support. The foam has adhered beneath the DRA using RTV silicone adhesive (with a dielectric constant of $\epsilon _{g}$ $\simeq$ 3), ensuring stable placement without significantly affecting the antenna’s performance.

The six P-I-N diodes are toggled ON and OFF states through a DC-biased circuit, as depicted in Fig. 1c. Each P-I-N diode has two DC bias lines. Each line comprises two 27 nH inductors (model: LQP03HQ27NH02D) connected in series, acting as RF chokes to present high impedance and block the RF signals from entering the DC bias lines. A 1.2 k$\Omega$ resistor is connected in series with the inductors to stabilize the biasing circuit. To ensure DC isolation, 0.1-mm-wide DC slotlines are etched into the metal strip adjacent to the dielectric resonator. The slots prevent direct DC-current flow while maintaining RF continuity. Twelve 100-pF capacitors (model: 251R14S101GV4T from Johanson Technology) are placed within the DC slots to couple RF signals across the antenna structure while isolating them from the DC circuitry. The diode bias lines are connected to the external DC power supplies via fine wires. The varactor diode capacitance is adjusted by varying the applied voltage, and the P-I-N diodes are activated or deactivated using controlled bias voltages, enabling dynamic reconfiguration of the antenna’s polarization states. Thus, the design ensures precise control over the diodes’ operation while minimizing interference between RF and DC circuits, thereby maintaining the antenna’s optimal performance.

### Experimental results

**Fig. 6:**
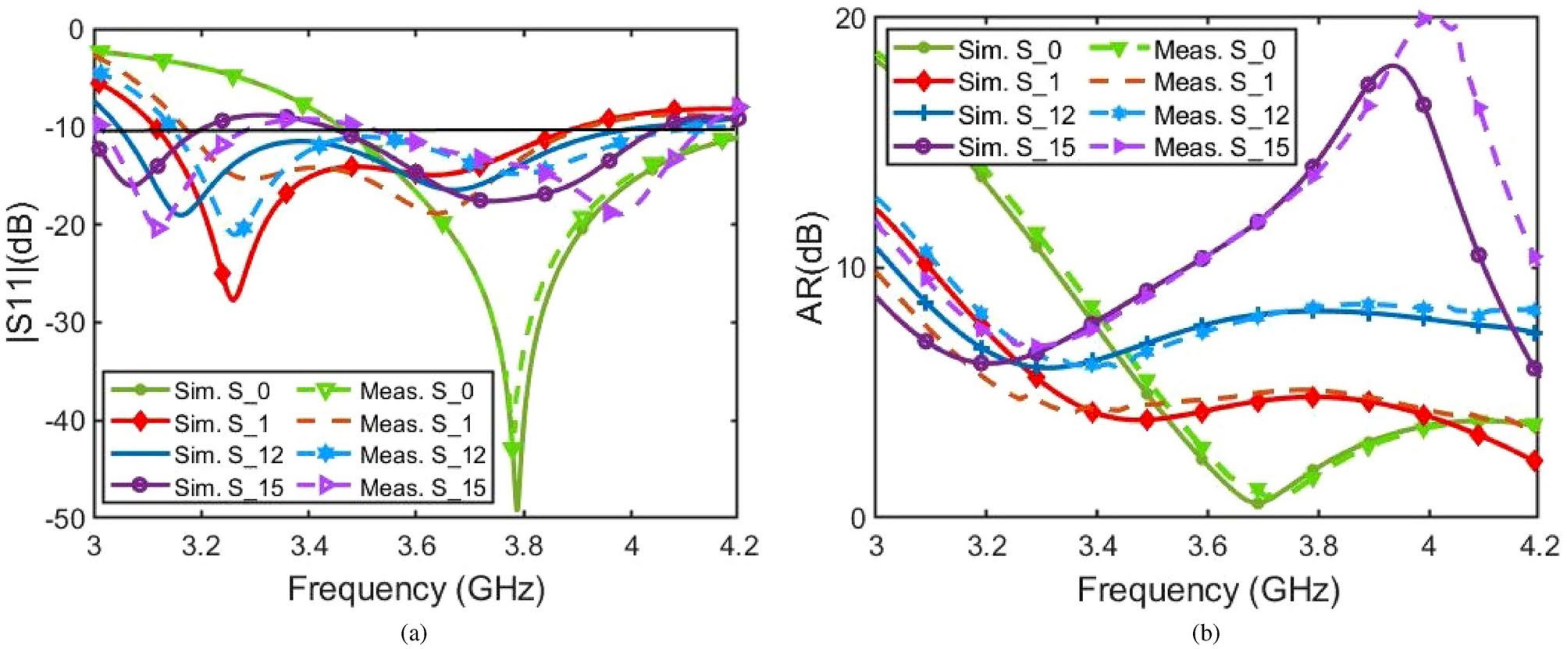
Simulated and measured (**a**) |*S*11|, and (**b**) Axial Ratio.

Fig. 6 presents the simulated and measured S-parameters (Fig. 6a) and axial ratio (AR) (Fig. 6b) of the proposed ADRA selected states. These states include when the varactor diode is set to 1.51 pF with three different states of PIN diodes: Zero refers to diode’s OFF, and 1 refers to diode’s ON, $S\_1$ (000000), $S\_12$(111000), and $S\_15$(111111) to modulate the AR and S-parameters, as well as when all varactor and P-I-N diodes are switched off $S\_0$. The results demonstrate a strong correlation between the simulated and measured data, with only minor discrepancies, likely due to fabrication tolerances and manual assembly imperfections. It is noted that using high-performance materials, such as ECCOSTOCK®  HiK dielectric, and integrating multiple diodes and precision biasing circuitry may increase the overall fabrication cost, limiting accessibility for cost-sensitive applications. Besides, the multilayer configuration with nine stacked dielectric layers increases the antenna’s overall height, which may restrict its use in applications requiring stringent size.

It is important to note that, due to the wide range of possible capacitor values and numerous available states, it is challenging to represent all of them in a single graph. Therefore, Table 3 provides a comprehensive overview of the antenna’s performance, detailing impedance bandwidth, AR, and tilt angle across 32 different states, reflecting changes in varactor capacitance and P-I-N diode activation.

**Fig. 7:**
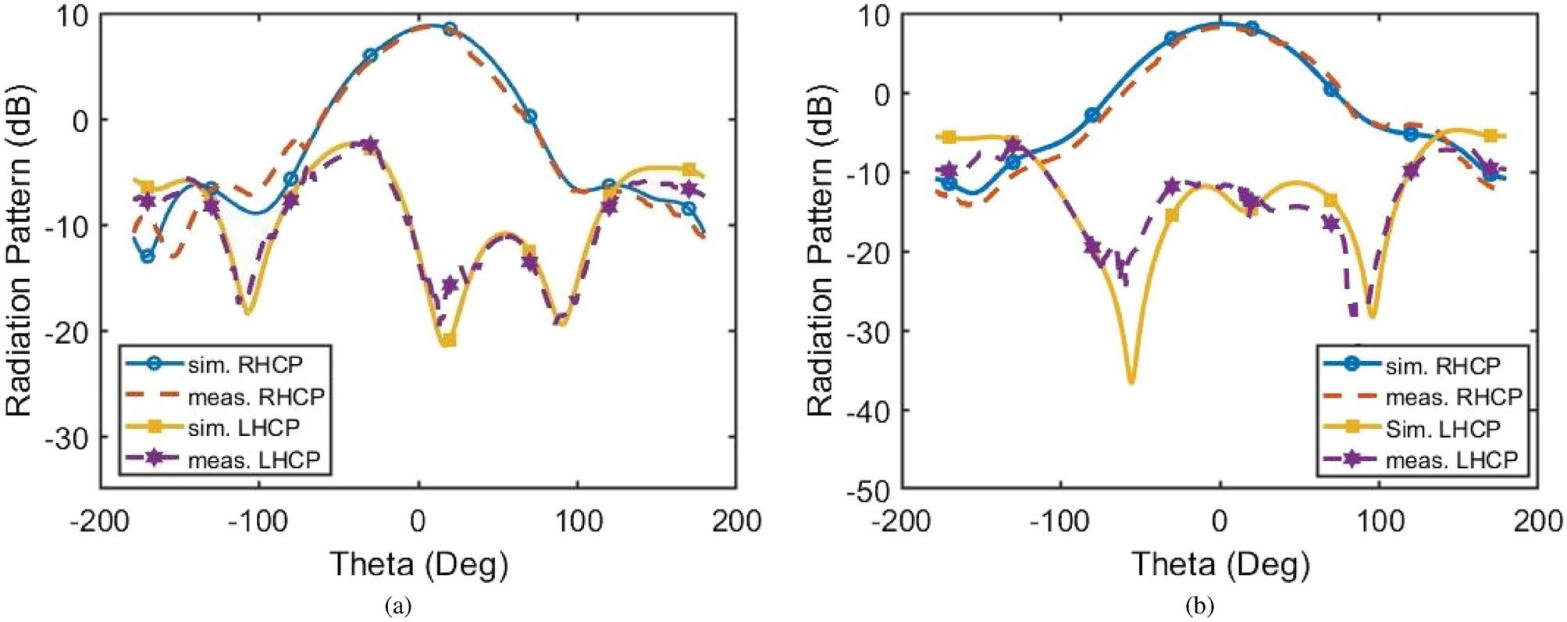
Simulated and measured RHCP and LHCP radiation patterns in (**a**) xz-plane and (**b**) yz-plane at 3.8 GHz.

As shown in Table 3, adjusting the varactor capacitance broadens the range of achievable states. Table 3 highlights how varying the varactor’s capacitance affects the electrical length of the strip. This change in capacitance mirrors the effect of physically altering the strip length, particularly on the reflection coefficient $|S_{11}|$. However, it also reduces the AR and tilt angle. In summary, the voltage variation across the varactor diode alters its capacitance, which impacts the effective electrical length of the strip-an established behavior in such designs. The measured results verify that decreasing the capacitance has an equivalent impact on bandwidth, as a reduction in physical length leads to a decrease in both AR and tilt angle. Fig. 7 depicts the simulated and measured LHCP and RHCP radiation patterns of the proposed antenna in the xz-plane $\phi = 0 ^{\circ }$ (Fig. 7a) and yz-plane $\phi = 90 ^{\circ }$ (Fig. 7b) at 3.8 GHz when varactor and all diodes are OFF. The simulated and measured patterns exhibit close agreement. Notably, the disparity between LHCP and RHCP radiation levels exceeds 18 dB, confirming the radiation’s purity. It is noted that the proposed antenna provides a total efficiency higher than 93$\%$ in the desired frequency bands, with a maximum gain of about 7.64 dBi for simulation and 7.09 dBi for measurements, respectively.

**Table 3:** All Possible States Using Varactor Diode with 2 Different Varactor Capacitances: 1: (C = 1.13 pF); 2: (C = 2.28 pF).

Status	0 = OFF, 1 = ON	V’s State	Simulated	Simulated	Simulated	Measured	Measured	Measured
			Tilt Angle	AR (dB)	BW (GHz)	Tilt Angle	AR (dB)	BW (GHz)
$S_{1}$	000000	1	88.34	4.76	3.12-3.9	88.76	4.43	3.35-4.12
		2	87.12	5.28	3.1-3.91	87.31	5.32	3.3-4.06
$S_{2}$	100000	1	86.74	5.09	3.1-3.91	86.74	5.19	3.36-4.22
		2	85.37	5.79	3.06-3.96	85.67	5.89	3.22-4.04
$S_{3}$	000100	1	88.49	5.34	3.1-3.92	87.21	5.63	3.4-4.21
		2	87.46	6.25	3.07-3.94	87.76	6.15	3.36-3.91
$S_{4}$	100100	1	87.18	5.66	3.08-3.92	86.78	5.86	3.2-3.98
		2	86.1	7.11	3.04-4.03	85.81	7.02	3.23-4.33
$S_{5}$	110000	1	84.58	5.62	3.08-3.96	84.05	5.43	3.22-3.98
		2	82.74	7.79	3.03-4.2	82.13	7.56	3.21-4.2
$S_{6}$	000110	1	90.22	6.35	3.08-3.94	89.81	6.46	3.28-4.05
		2	91.27	8.38	3.03-4.02	90.97	8.04	3.18-4.17
$S_{7}$	110100	1	85.44	6.19	3.06-4.2	86.07	6.38	3.31-4.2
		2	85.28	9.44	3.06-4.2	85.63	9.88	3.29-4.2
$S_{8}$	100110	1	89.27	6.66	3.06-3.96	89.09	7.11	3.24-4.13
		2	90.9	9.6	3-4.1	89.92	10.12	3.2-4.16
$S_{9}$	000111	1	94.07	7.6	3.07-3.95	93.87	7.55	3.31-4.11
		2	100.95	11.18	3.01-3.95	101.46	10.83	3.19-4.04
$S_{10}$	100111	1	93.4	7.89	3.06-3.96	93.61	8.03	3.23-4.12
		2	101.43	12.57	3.39-3.96	100.88	13.17	3.45-4.2
$S_{11}$	110110	1	88.05	7.215	3.05-4.2	88.1	7.44	3.2-4.2
		2	91.92	12.83	3.39-4.14	92.13	13.36	3.40-4.12
$S_{12}$	111000	1	82.49	6.67	3.06-4.2	82.73	82.73	3.28-4.2
		2	87.2	14.56	3.52-4.12	86.93	14.02	3.56-4.06
$S_{13}$	110111	1	92.54	8.44	3.04-4.02	90.98	9.04	3.21-4.16
		2	103.8	15.79	3.40-3.97	103.2	15.03	3.48-4.09
$S_{14}$	111100	1	83.81	7.25	3.04-4.2	83.39	7.91	3.35-4.2
		2	91.97	16.89	3.48-4.08	91.62	16.02	3.52-3.98
$S_{15}$	111111	1	91.8	9.56	3.02-4.19	92.21	10.11	3.21-4.2
		2	111.08	20.72	3.41-3.98	110.68	21.07	3.46-3.97
$S_{16}$	111110	1	86.93	8.27	3.03-4.19	87.09	9.67	3.22-4.2
		2	100.61	21.19	3.43-4.05	100.09	22.4	3.49-4.08

**Table 4:** All possible states using varactor diode with 2 different varactor capacitances: 1: (C = 1.13 pF); 2: (C = 2.28 pF).

Status	0 = OFF, 1 = ON	V’s State	E_v_/E_h_	$\delta _L$
$S_{0}$	000000	OFF	1.3784	93.7047
$S_{1}$	000000	1	1.7297	88.1221
		2	1.8288	86.2613
$S_{2}$	100000	1	1.7928	85.9229
		2	1.9279	83.3854
$S_{3}$	000100	1	1.8468	87.9530
		2	2.0450	86.0921
$S_{4}$	100100	1	1.9099	86.0921
		2	2.2432	82.8779
$S_{5}$	110000	1	1.8829	82.5395
		2	2.3423	75.6036
$S_{6}$	000110	1	2.0811	90.3213
		2	2.6216	92.8589
$S_{7}$	110100	1	2.0090	83.0470
		2	2.8829	77.8028
$S_{8}$	100110	1	2.1532	88.7988
		2	3.0180	92.3514
$S_{9}$	000111	1	2.3604	97.9339
		2	2.9730	121.9560
$S_{10}$	100111	1	2.4595	97.0881
		2	3.2252	127.8769
$S_{11}$	110110	1	2.2883	86.4304
		2	4.3333	97.9339
$S_{12}$	111000	1	2.0811	77.6336
		2	5.1712	75.9419
$S_{13}$	110111	1	2.6216	95.7347
		2	3.3964	144.2863
$S_{14}$	111100	1	2.2432	78.6486
		2	6.7928	103.1782
$S_{15}$	111111	1	2.9910	94.7197
		2	2.5225	164.5866
$S_{16}$	111110	1	2.5676	83.2162
		2	4.8378	154.0981

**Fig. 8:**
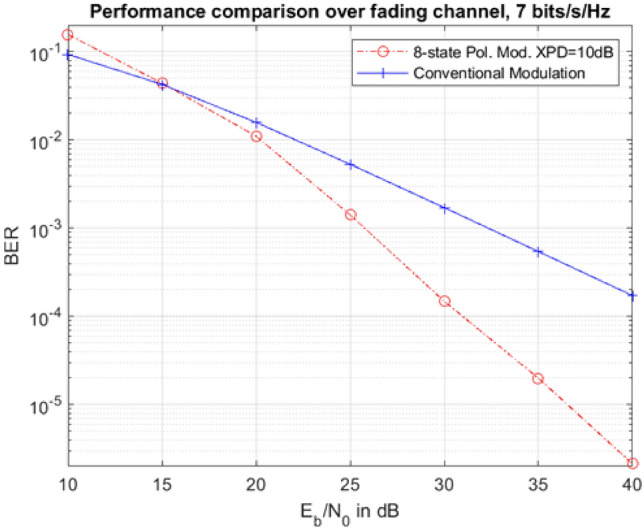
BER performance comparison between polarization and conventional modulation.

**Fig. 9:**
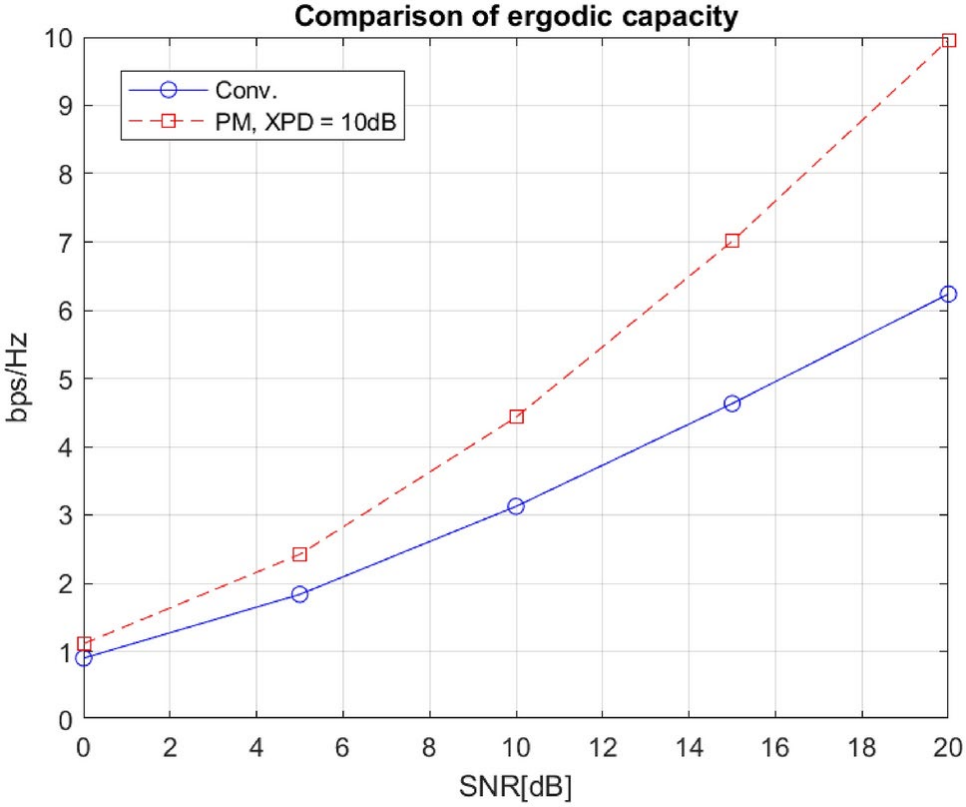
Ergodic capacity comparison.

Figures 8 and 9 depict the system-level performance gain for our designed polarization reconfigurable DRA. In particular, Fig. 8 presents a performance comparison between the proposed polarization modulation and a conventional modulation scheme, both achieving the same spectrum efficiency of 7 bits/s/Hz. In the former case, 128QAM is employed, while in the latter case, the cross-polar discrimination (XPD) is set to 10dB to simulate practical scenarios. We use 8-state polarization modulation, representing 3 bits of information, combined with 16QAM modulation, contributing 4 bits per symbol. The selected 8 states, including S2, S9, S10, and S12-S16, shown in Table 4, are chosen without specific optimization of distance distribution among them. Despite this, the polarization modulation achieves approximately a 10dB gain at a target BER of $10^{-4}$, demonstrating its superiority over the conventional scheme.

In Fig. 9, it is evident that the proposed system achieves significantly higher ergodic capacity compared to the conventional system. For example, at SNR=16dB, the capacity of the proposed system is around 7.5 bits/s/Hz/antenna, representing a 50$\%$ improvement in spectrum efficiency compared to the conventional system. This paper shows that ADRA design achieves better BER performance (see Fig. 8) and higher ergodic capacity (see Fig. 9) by efficiently using polarization states to adapt to channel conditions, leading to maximized transmission rate. Table 5 compares the performance of the proposed antenna with other reported polarization-reconfigurable DRAs, considering parameters such as polarization states, tilt-angle reconfigurability, size, gain, bandwidth, and simulated efficiency. Notably, the proposed antenna is the only design offering tilt-angle reconfigurability and continuous polarization agility, ranging from circular polarization (CP) to nearly linear polarization.

**Table 5:** Comparison between the proposed antenna and some reported similar antennas.

Reference	Polarization	Tilt Angle	Size	Gain	Bandwidth	Sim. Efficiency
^25^	Three states	NO	140*140*32.87	2.26–2.51	18.75	77–99
	Omnidirectional				4.5	
	To broadside					
^26^	Two states	NO	80*80*25.5	2–6	35.5	80
	Broadside					
	To conical					
^27^	Five states	NO	110*110*22	3.5–6.6	36.8	80
	Vertical and $\pm 45^\circ$					
	RHCP to LHCP					
^28^	Two states	NO	120*120*30	5–5.5	35.6	70
	RHCP to LHCP					
^29^	Three states	NO	140*140*23.4	6	18	80–90
	y-axis and $\pm 45^\circ$					
^30^	Three states	NO	N/A	5.4–6.2	15	65–79
	y-axis and $\pm 45^\circ$					
Proposed	Continuous	YES	100*100*10.5	7.09	9.3	93
Antenna	LP-eliptical and CP					

## Conclusion

A novel polarisation reconfigurable anisotropic dielectric resonator antenna has been presented, showcasing the ability to tune both the axial ratio and tilt angle. We have proposed a new modulation scheme to leverage this antenna design and harness the additional degrees of freedom in the polarization domain. This scheme utilizes polarization, tilt angle, and axial ratio to convey supplementary information. The fabricated reconfigurable structure undergoes measurement, revealing that the antenna performances closely align with the simulation results, demonstrating an excellent agreement. The antenna achieves a measured gain of approximately 7.09 dBi at 3.8 GHz, with the impedance matching bandwidth measured from 3.56 to 3.91 GHz, fully overlapping across all polarization states. The proposed method demonstrates significantly enhanced performance, providing a promising solution for boosting the capacity of future wireless systems and achieving high data transmission rates. The ability to dynamically alter polarization states is crucial in mitigating multipath interference and signal fading due to atmospheric conditions. With its reconfigurability, the ADRA is well-suited for applications requiring high reliability in environments with fluctuating signal paths and challenging conditions.

## Data Availability

The datasets used and/or analysed during the current study are available from the corresponding author on reasonable request.
